# Assessing the sweet sorghum-based ethanol potential on saline–alkali land with DSSAT model and LCA approach

**DOI:** 10.1186/s13068-021-01896-z

**Published:** 2021-02-16

**Authors:** Jingying Fu, Xiaoxi Yan, Dong Jiang

**Affiliations:** 1 State Key Laboratory of Resources and Environmental Information System, Institute of Geographic Sciences and Natural Resources Research, Chinese Academy of Sciences, 11A Datun Road, Beijing, 100101 China; 2 College of Resource and Environment, University of Chinese Academy of Sciences, No. 19A Yuquan Road, Beijing, 100049 China; 3 Key Laboratory of Carrying Capacity Assessment for Resource and Environment, Ministry of Natural Resources, 11A Datun Road, Beijing, 100101 China; 4 Department of Geography and Resource Management, The Chinese University of Hong Kong, Hong Kong, China

**Keywords:** Biomass, Energy–food nexus, Sweet sorghum-based ethanol, Life cycle assessment, The DSSAT model; saline–alkali land

## Abstract

**Background:**

The key problem of non-grain energy plants’ scale development is how to estimate the potential of GHG emission reduction accurately and scientifically. This study presents a method coupled DSSAT (the Decision Support System for Agrotechnology Transfer) and the life cycle assessment (LCA) method to simulate the spatial distribution of sweet sorghum-based ethanol production potential on saline–alkali land. The GHG (greenhouse gas) emission mitigation and net energy gains of the whole life of sweet sorghum-based ethanol production were then analyzed.

**Results:**

The results of the case study in Dongying, Shandong Province, China showed that developing sweet sorghum-based ethanol on saline–alkali land had GHG emission mitigation and energy potentials. The LC-GHG emission mitigation potential of saline–alkali land in Dongying was estimated at 63.9 thousand t CO_2_ eq, equivalent to the carbon emission of 43.4 Kt gasoline. The LC-NEG potential was predicted at 5.02 PJ, equivalent to the caloric value of 109 Kt gasoline. On average, LC-GHG emission mitigation and LC-NEG were predicted at 55.09 kg CO_2_ eq/t ethanol and 4.33 MJ/kg ethanol, respectively.

**Conclusions:**

The question of how to evaluate the potential of sweet sorghum-based ethanol development scientifically was solved primarily in this paper. The results will provide an important theoretical support for planning the bioenergy crops on saline–alkali land and develop the fuel ethanol industry.

## Background

Biofuels have received much attention in recent years because of their energy and environmental efficiencies, with hopes to alleviate the energy crisis and prevent further climate change. Biomass has garnered tremendous interests as a potential feedstock for clean energy production at the same time [1, 2]. The conversion of sugar and starch to ethanol has been demonstrated on an industrial scale, for example in Brazil and the United States, and the ethanol produced has proved competitive with conventional gasoline because of various incentives [3]. However, conflicts between biofuel feedstocks and food crops still exist [4]. In China, the government claimed that developing biofuels should not cause conflicts with food security. “Not using the grain intended for food, and not occupying the lands intended for grain production” is a leading principle for biofuel development. Also, China encourages people to use marginal lands with little agricultural value for biofuel development. Saline–alkali land is an important marginal land type. The large area of saline–alkali land poses a serious threat to regional agricultural development. The reason is that salinization causes detrimental effects on crop growth and yield, damages to infrastructure, reduction in water quality, sedimentation problems, and finally soil erosion when the vegetation is too strongly affected by the amount of salts [5–7]. Even though a series of methods have been applied for saline–alkali land amendment, the problem of salinization is still a serious soil degradation problem at home and abroad [8–10].

Sweet sorghum is selected as the study object for two reasons. First, studies showed that planting sweet sorghum on saline–alkali land could be helpful to maintain the sustainable development of the land [11]. It can also help to improve the sustainable production of soil and other crop systems [12–14]. Second, sweet sorghum has attracted both the government’s and farmer’s attention as a non-food feedstock for bio-ethanol [15, 16]. Sweet sorghum has high stress resistance to severe environments such as soil salinization and poor soil properties. It has lower environment requirements than other crops like cotton and maize [17]. Moreover, sweet sorghum stalks hold high levels of sugar content which is very important for bio-ethanol production [18, 19]. Also, the energy output of sweet sorghum is possible to increase with the further technical development [17].

In recent years, literature on the sweet sorghum-based ethanol has increased, with most of the studies integrating LCA and DSSAT methods, and the related works have being widely carried out in China. Hao et al. [20] used DSSAT model and GIS technology to conduct comprehensive evaluation research on water stress of sweet sorghum fuel ethanol on a national scale, and determined the direction of sustainable development of sweet sorghum region. Yan et al. [21] presented an integrated method of assessing sweet sorghum-based ethanol potential in China in compliance with the Water–Energy–Food nexus principles. The spatial distribution of water consumption, net energy gain, and Greenhouse Gas emission reduction potentials of developing sweet sorghum-based ethanol on marginal lands instead of cultivated land in China were discussed. However, research into the assessment of the non-grain-based fuel ethanol potential at the regional level is still relatively limited and needs to be improved, since there are distinct differences of growing conditions and environmental features between different regions in China. Under these circumstances, this study was focused on how to evaluate the LC-GHG (Life Cycle Assessment of Greenhouse Gas) emission mitigation and LC-NEG (Life Cycle Assessment of Net Energy Gain) potentials of developing sweet sorghum-based ethanol on saline–alkali land, which provides a new idea to deal with the conflicts between bio-ethanol feedstocks and food crops. A case study was carried in Dongying, Shandong Province, China to test the feasibility of this idea.

### Study area

Dongying, Shandong Province, China, was selected as the study area (see Additional file 1: Appendix A). The city is located on the banks of the Yellow River Delta and has a total area of 7923 square kilometers (3059 sq. mi). Dongying City was established in October 1983, consisting of 3 districts and 2 counties, including Dongying District, Hekou District, Kenli District, Guangrao County, and Lijin County. The climate in Dongying is between the humid continental and humid subtropical regimes.

Saline–alkali soil is one of the most critical problems in Dongying. The mild-to-moderate saline soil with soil conductivity greater than 12.42 μS⋅m^(-1) in Dongying City accounts for 52% of the land area in Dongying City, accounting for 418,700 hm^2^ [22]; the soil pH in Dongying City is basically between 8.09 and 8.35, and the pH of the heavily saline soil is above 8.5 [23]. In summary, Dongying is a typical sea city with large area of saline–alkali soil [24].

## Results

### Estimation of sweet sorghum biomass and ethanol yield

**Fig. 1:**
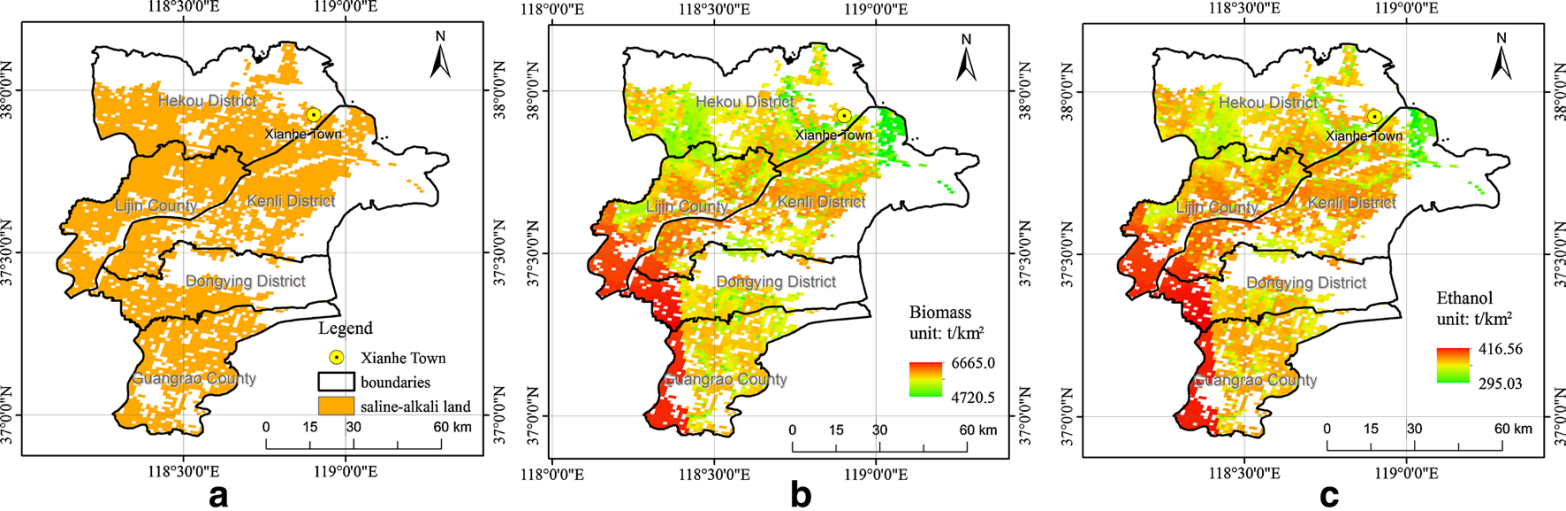
Spatial distribution of saline–alkali land (**a**), sweet sorghum biomass (**b**), and ethanol yield (**c**)

An estimation of sweet sorghum biomass was conducted on saline–alkali land in Dongying using the GIS-based DSSAT model. Except for developed land, water bodies, or other landscapes that could not be used for sweet sorghum cultivation, the area of saline–alkali land in Dongying was 4261 km^2^ (see Fig. 1a). The spatial distribution of sweet sorghum biomass is shown in Fig. 1b, and the statistics of yields are shown in Table 1. According to Tian et al. and Wang et al. [25, 26], the conversion rate of sweet sorghum yield to ethanol fuel production is 16:1, which means that it takes 16 kg sweet sorghum to produce 1 kg bio-ethanol, and the spatial distribution of ethanol is shown in Fig. 1c.

**Table 1:** Statistics of sweet sorghum biomass and ethanol yield of each county and district

Name	Area (km^2^)	Saline–alkali area (km^2^)	Percentage of saline–alkali land	Sweet sorghum biomass(t)	Biomass yield (t/km^2^)	Ethanol (t)
Guangrao County	1213	770	63%	4,732,561	6146.18	295,785
Dongying District	1201	489	41%	3,017,733	6171.23	188,608.3
Lijin County	1111	890	80%	5,473,609	6150.12	342,100.6
Kenli District	2270	1143	50%	6,890,954	6028.83	430,684.6
Hekou District	2128	969	46%	5,703,120	5885.57	356,445
Summary	7923	4261	54%	25,817,976	6059.14	1,613,623

According to Table 1, the highest yield per km^2^ was obtained in the Dongying District, whereas the lowest yield per km^2^ was in the Hekou District. To summarize, the simulated yield of sweet sorghum was 25.82 million tons on the saline–alkali land in Dongying City, and the weight of ethanol was 1.61 million tons.

### LC-GHG emission mitigation assessment results

**Fig. 2:**
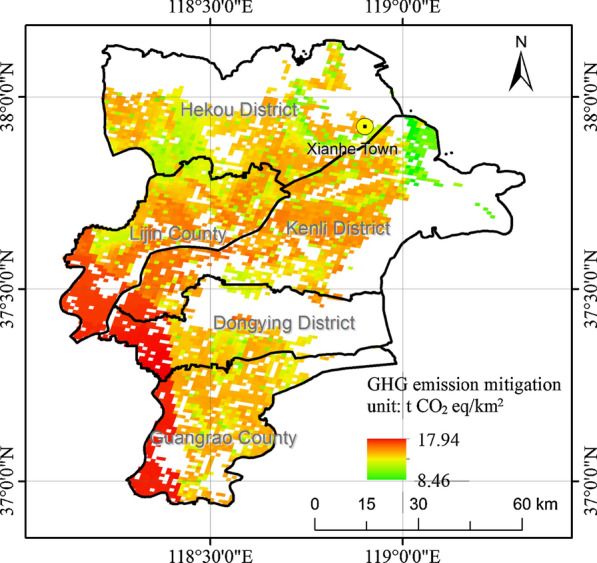
Spatial distribution of sweet sorghum-based ethanol GHG emission mitigation

**Fig. 3:**
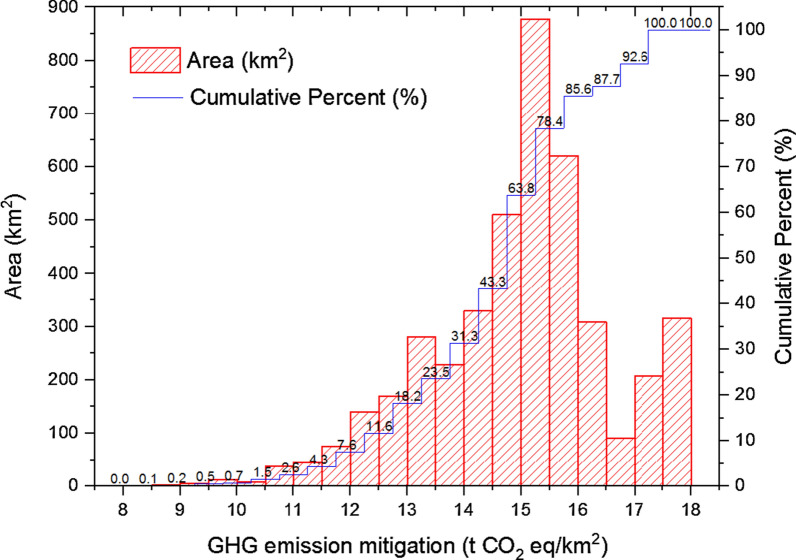
Histogram of LC-GHG emission mitigation of sweet sorghum-based ethanol (data for this histogram, see Additional file 1: Appendix B)

According to life cycle inventory analysis, the spatial distribution of sweet sorghum-based ethanol GHG emission mitigation is shown in Fig. 2, and the histogram is shown in Fig. 3.

According to Fig. 2, Dongying showed GHG emission mitigation potential by developing sweet sorghum-based ethanol on the saline–alkali land. The value of GHG emission mitigation results ranged from 8.46 to 17.94 t CO_2_ eq/km^2^. High-value areas were located in the southeast region of the city, whereas low-value areas were located near the coastal. The summary of GHG emission mitigation potential of saline–alkali land in Dongying was predicted at 63.9 thousand t CO_2_ eq.

According to Fig. 3, 90% of saline–alkali land has a GHG emission mitigation value of 12 t CO_2_ eq/km^2^ or more. Group with values ranging from 15 to 15.5 t CO_2_ eq/km^2^ took the first position in all the groups, and occupied about 15% of the saline–alkali land in Dongying.

In conclusion, developing sweet sorghum-based ethanol on saline–alkali land in Dongying is predicted to be helpful from the perspective of GHG emission mitigation potential.

### LC-NEG assessment results

**Fig. 4:**
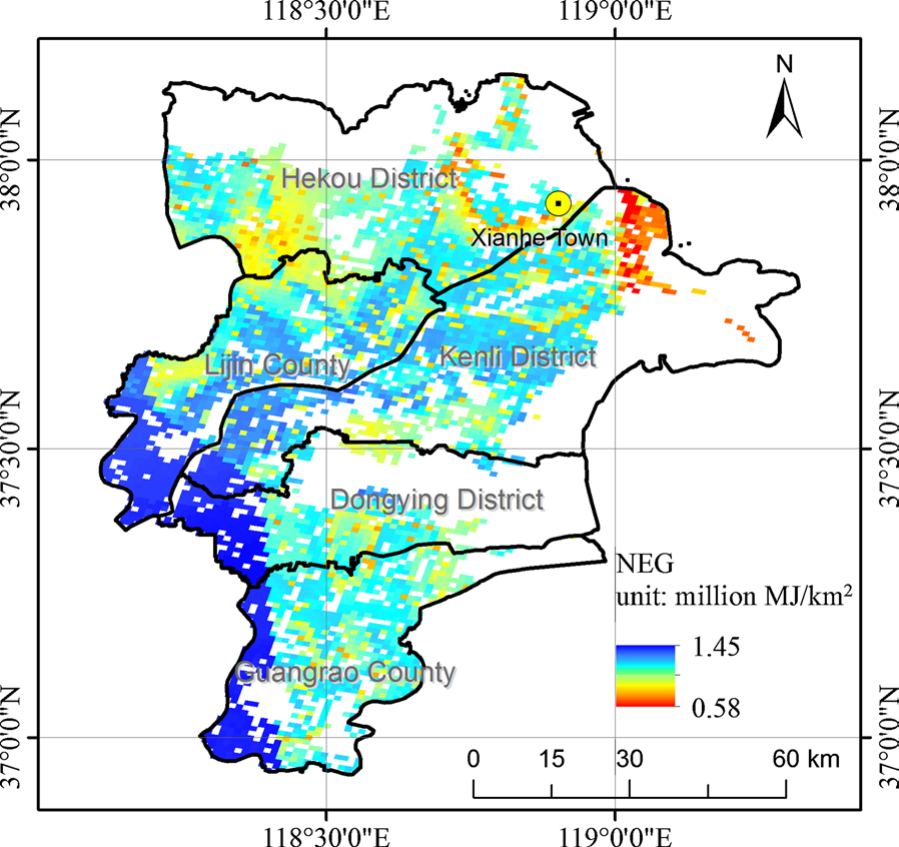
Spatial distribution of LC-NEG assessment result of sweet sorghum-based ethanol

**Fig. 5:**
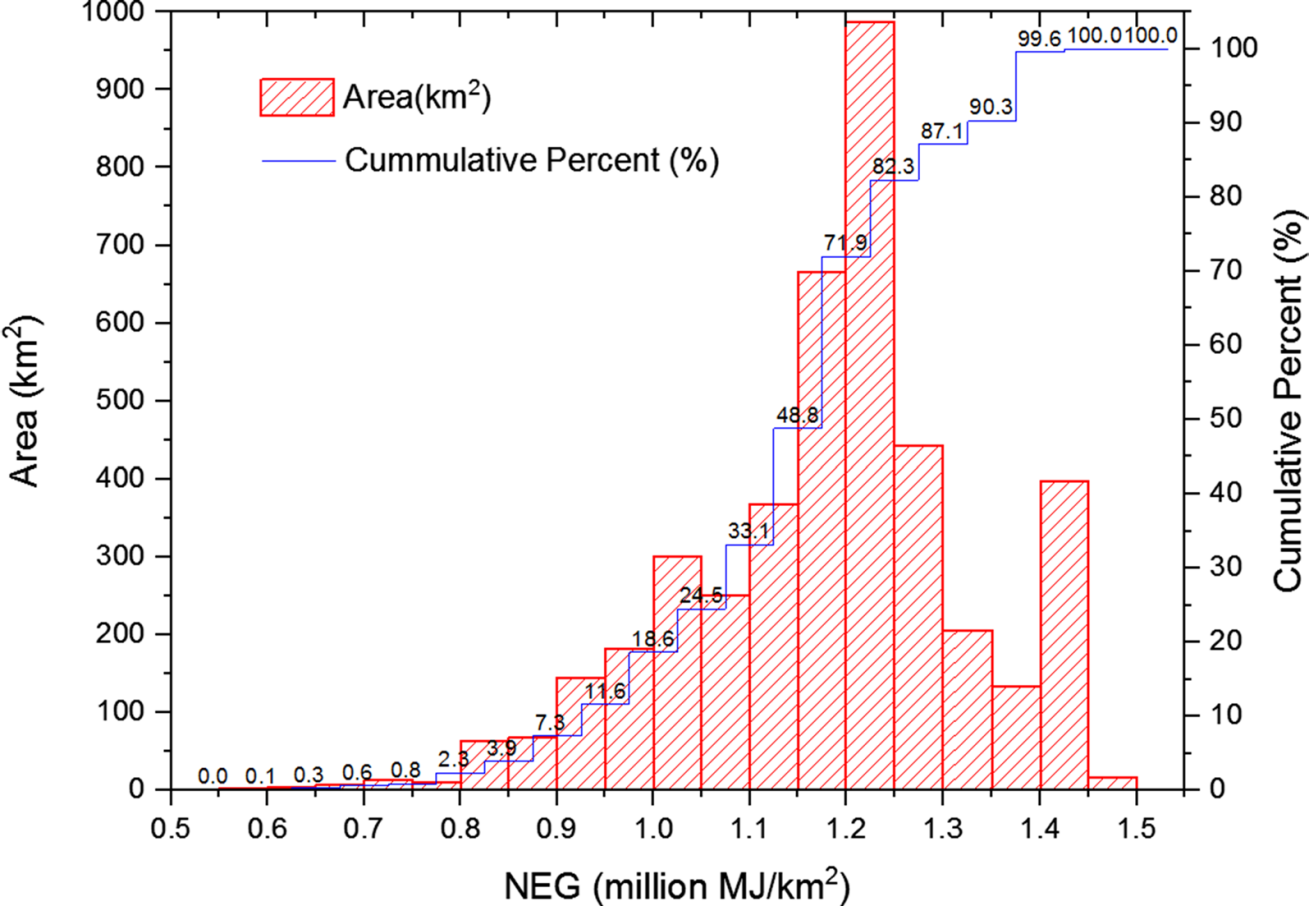
Histogram of LC-NEG of sweet sorghum-based ethanol (data for this histogram, see Additional file 1: Appendix C)

LC-NEG assessment was calculated based on formulas in Section “LC-NEG assessment” and datasets in “Inventory analysis of LC-NEG assessment”. The spatial distribution of LC-NEG assessment result of sweet sorghum-based ethanol is shown in Fig. 4, and the histogram is shown in Fig. 5. The statistics of LC-NEG in every district and county in Dongying were shown in Table 2.

**Table 2:** Statistics of the saline–alkali land area and NEG of sweet sorghum-based ethanol of each county and district

Name	Saline–alkali land area (km^2^)	NEG (10^6^ MJ)	Average NEG (10^6^ MJ/ km^2^)
Guangrao County	770	937.20	1.22
Dongying District	489	600.67	1.23
Lijin County	890	1084.83	1.22
Kenli District	1143	1331.11	1.16
Hekou District	969	1066.28	1.10
Summary	4261	5020.10	1.18

According to Figs. 4 and 5, NEG values ranged from 0.58 to 1.45 million MJ/km^2^ on saline–alkali land in Dongying. High-value areas were located in the southeastern part, whereas low-value places were centralized near the coastal areas. Places with NEG values ranging from 1.2 to 1.25 million MJ/km^2^ held the largest saline–alkali areas with a percentage of above 20%, followed by the places with NEG values ranging from 1.15 to 1.2 million MJ/km^2^.

According to Table 2, the NEG potential of developing sweet sorghum-based ethanol in saline–alkali land in Dongying was predicted at 5020.10 million MJ every year. Kenli County was estimated to get the highest NEG potential in total, whereas Dongying District was predicted to get the highest average NEG potential followed by Guangrao County and Lijin County.

In conclusion, from the perspective of NEG potential, developing sweet sorghum-based ethanol could be an alternative utilization of the saline–alkali land.

## Discussions

### Comparison with other studies

In this study, a new attempt of developing sweet sorghum-based ethanol on saline–alkali land was conducted, and two types of bio-ethanol potentials, LC-NEG potential and LC-GHG emission mitigation potential, were assessed in the case study in Dongying, Shandong Province, China. The results were compared with other previous studies in this field.

Wang et al. [27] conducted an environmental sustainability study of sweet sorghum stem-based ethanol on saline–alkali land. The LC-NEG was predicted at 17.21 MJ/L ethanol. In this study, the LC-NEG ranged from 0.58 to 1.54 million MJ/km^2^, whereas the bio-ethanol ranged from 295.03 to 416.56 t/km^2^. We know that the density of ethanol is 0.789 kg/L. Thus the range of LC-NEG in this study was calculated as 2.49–4.69 MJ/L ethanol which was much lower than the value predicted by Wang et al. [27]. The reason resulting in the difference is that Wang et al. [27]’s study applied fewer items of energy input, which seem to be inadequate for LC-NEG assessment, than the items applied in this study. For example, at the bio-ethanol conversion stage, a very important stage over the life cycle of bio-ethanol, Wang et al. [27]’s study calculated only two energy input items (electricity and auxiliary materials), whereas our study calculated four energy input items (including electricity, coal, steam, and other inputs). Thus, the energy input at the bio-ethanol conversion stage was calculated as 0.78 MJ/L ethanol in Wang et al. [27]’s study and 19,123.15 MJ/kg ethanol, equivalent to 24.23 MJ/L ethanol in this paper, respectively. Besides, Wang et al. [27] took the saline–alkali land as a uniform region with the same input and output, whereas this study assessed LC-NEG at a spatial scale regarding the different growing conditions for sweet sorghum. Compared with cassava, sweet sorghum showed much advance from the perspective of GHG emission. The study of Numjuncharoen et al. [28] estimated that the GHG emission of cassava ranged from 0.548 to 1.097 kg CO_2_ eq/L ethanol which was equivalent to 0.16 to 0.65 kg CO_2_ eq/L ethanol of GHG emission mitigation compared with gasoline. In this study, the total LC-GHG emission mitigation and ethanol yield were predicted at 63.9 thousand t CO_2_ eq and 1.16 t ethanol, respectively. The average LC-GHG emission mitigation potential could be calculated at 0.043 kg CO_2_ eq/L ethanol which was much lower than the potential of cassava. However, growing cassava has a high requirement of temperature, whereas sweet sorghum does not. Thus, different types of species should be planted for different regions.

### Measures for improvements

Two aspects could be considered for a better performance of the assessment. First, the DSSAT model could be calibrated with sugar content records of sweet sorghum, so we can apply a more detailed bio-ethanol potential assessment, because sugar content in the stems has considerable influence on bio-ethanol yield. In addition, sugar content might be affected by the growing environment of sweet sorghum. In this study, we used a constant conversion value of sweet sorghum to bio-ethanol. However, sweet sorghum with low quality such as low sugar content would not meet the requirement. Second, different energy crops should be applied to a comprehensive assessment. Although sweet sorghum-based ethanol production showed potential on saline–alkali land in Dongying, it does not mean that sweet sorghum is the best species for bio-ethanol development in this region. Besides, there are many kinds of marginal land types from home and abroad. One species cannot adapt to all land types, so more species should be studied to support policymakers.

## Conclusions

Under the requirements of food security, this study proposed an idea of developing bio-ethanol on saline–alkali land which has low efficiency in growing crop plants. The case study in Dongying, Shandong province, China showed that developing sweet sorghum-based ethanol on saline–alkali land is feasible. It is possible to achieve a reduction in GHG emission and net energy gains.

Results showed that saline–alkali land in Dongying covered an area of 4261 km^2^, occupying 53.78% of the entire city. Developing sweet sorghum-based ethanol presented the potential of getting 1.16 million tons of ethanol on saline–alkali land. The result of LC-GHG emission mitigation assessment showed that the potential of GHG emission mitigation in Dongying was 63.9 thousand t CO_2_ eq, equivalent to the carbon emission of 43.4 Kt gasoline. On average, per ton of sweet sorghum-based ethanol’s LC-GHG emission mitigation potential was estimated at 55.09 kg CO_2_ eq/t ethanol. According to the result of LC-NEG assessment, the potential of NEG on saline–alkali land in Dongying was 5020 million MJ, equivalent to the caloric value of 109 Kt gasoline. The average LC-NEG potential was predicted at 4.33 MJ/kg ethanol.

In conclusion, the saline–alkali land in Dongying could gain benefits by developing sweet sorghum-based ethanol. Making use of saline–alkali land can be a feasible alternative for bio-ethanol development. Further studies can be focused on selecting the dominated energy crops for different kinds of land-use types.

## Materials and methods

### System boundary

**Fig. 6:**
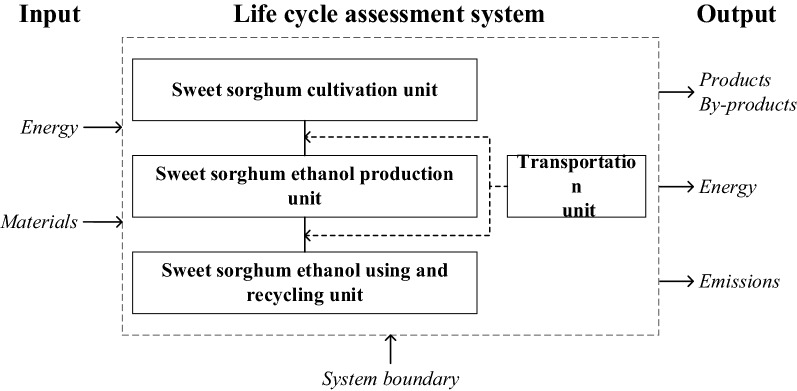
System boundary of LCA of sweet sorghum-based ethanol production, including the input to the system consisted energy and materials, and the output from the system included products/by-products, energy, and emission wastes

The goal of this study was to assess NEG (Net Energy Gain) and GHG (Greenhouse Gases) emission mitigation potentials of developing sweet sorghum-based ethanol on saline–alkali land. The system was composed of five units: sweet sorghum cultivation unit, bio-ethanol production unit, bio-ethanol combustion unit, and two transport units. The input to the system consisted of energy and materials, and the output from the system included products/by-products, energy, and emission wastes (e.g., Greenhouse Gases) into the environment. A schematic of system boundary is shown in Fig. 6.

### Development of the GIS-based DSSAT model

**Fig. 7:**
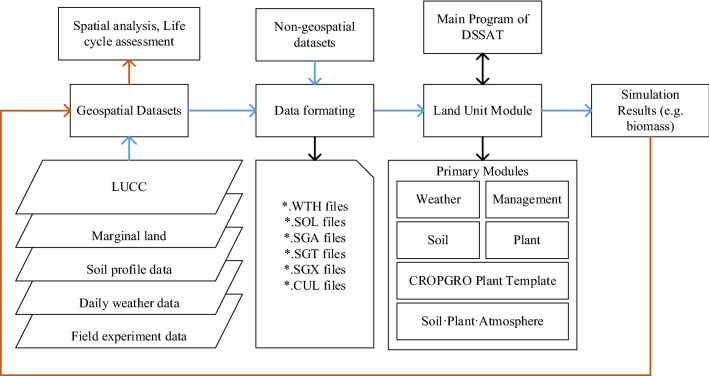
GIS-based DSSAT model for sweet sorghum biomass estimation. Solid blue arrows show the data input process of the GIS-based DSSAT model, whereas solid orange arrows show the data output process of the model

In this study, the DSSAT model was expanded to a spatial scale [29], as shown in Fig. 7. GIS was coupled with the DSSAT model through the Land Unit Module of the DSSAT platform. The input data of the extended DSSAT model include geographic data and non-geographical attribute data. The geographic datasets include LUCC (land use and cover change) data, marginal land data, daily meteorological data and soil profile data, etc. The attribute datasets mainly refer to crop species parameter data. The spatial data set was organized through spatial analysis technology and life cycle assessment methods, and the spatial datasets and non-spatial attribute datasets were transformed into the input data format (likely, *.WTH file, *.SOL file, *.SGA file, *.SGT file, *CUL file). Finally, the spatial simulation of sweet sorghum biomass and evapotranspiration was carried out through the DSSAT model, which was mainly realized through the land unit module of the DSSAT model, which mainly includes the meteorological module, the management module, the soil module, the planting module, and the CROPGRO crop module (CROPGRO is the module name) and the soil-crop-atmosphere module. Biomass of sweet sorghum was simulated by the GIS-based DSSAT model, because biomass of sweet sorghum was the raw material to produce bio-ethanol. Biomass (also dry mass or dry matter) is different from fresh mass. The amount of biomass is a measurement of the mass of the plant when completely dried. The biomass of sweet sorghum consisted of all its constituents excluding water.

### Life cycle assessment

**Fig. 8:**
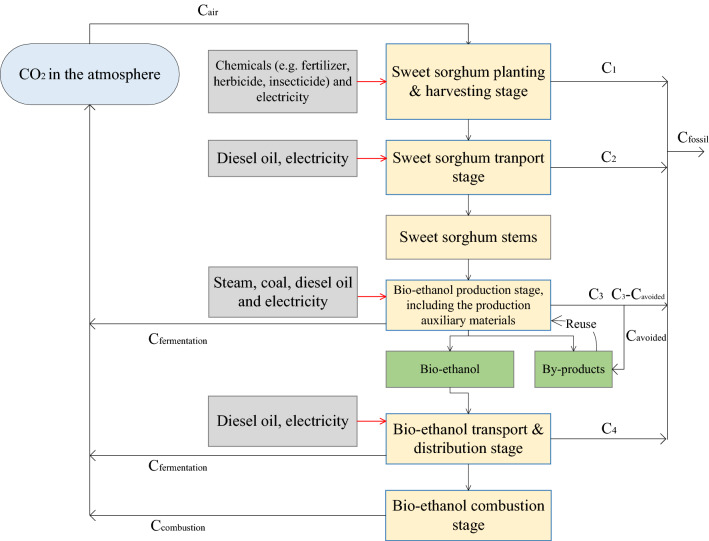
Life cycle inventory analysis of sweet sorghum-based ethanol. Yellow boxes show the six life cycle stages of sweet sorghum-based ethanol; gray boxes present the inventories of GHG emission mitigation and NEG assessment; and black arrows demonstrate the carbon cycle of sweet sorghum-based ethanol

The life cycle of the sweet sorghum-based ethanol was divided into five stages shown as blue boxes in Fig. 8, which begins with sweet sorghum planting procedure on saline–alkali soil to obtain the raw material for ethanol production. After the harvest, sweet sorghum stems are transported to ethanol factories in which products and by-products are manufactured through a series of industrial technological processes. Then, the ethanol is distributed to different facilities for use, and by-products are recycled to replace fuels with the same application. Ultimately, at the end of the life cycle, ethanol is combusted as biofuel.

For life cycle inventory analysis, different stages relate to diverse data items. Data items relating to NEG and GHG emission mitigation assessment are listed in Fig. 8 in gray boxes showing the process of life cycle inventory analysis in this paper. At the sweet sorghum planting stage, data on seeds, chemical fertilizer, pesticides, planting management details, harvest, and purchasing price of sweet sorghum stem are all required. At the stage of ethanol production, every industrial production process of ethanol production must be clearly identified and analyzed. Additionally, by-products should also be considered, because some are recycled or used to replace fossil fuels in the same application.

### LC-NEG assessment

NEG (Net Energy Gain) refers to an energy economics concept which is defined as calculating the difference between the energy output of an energy source and the total energy input to produce the energy source. In the life cycle assessment of sweet sorghum-based ethanol, LC-NEG assessment is defined as calculating the difference between the energy output of sweet sorghum-based ethanol combustion and the energy input of fossil fuels over the life cycle of sweet sorghum-based ethanol production.

According to Fig. 8, LC-NEG assessment included 1 stage (bio-ethanol combustion stage) of energy output, 4 stages (sweet sorghum planting and harvesting stage, sweet sorghum transport stage, bio-ethanol production stage, and bio-ethanol transport and distribution stage) of energy inputs, and 1 sub-stage of saving energy by using by-products. Thus, the formula of NEG is represented as follows [30, 31]:

$$ {\text{NEG }} = {\text{ BE}} - \left( {{\text{FE}}_{1} + {\text{FE}}_{{2}} + {\text{FE}}_{{3}} + {\text{FE}}_{{4}} - {\text{FE}}_{{{\text{by}}}} } \right), $$

where NEG is net energy gain; BE is energy output of bio-ethanol; FE_i_ (*i* = 1,2,3,4) are energy inputs of sweet sorghum planting and harvesting stage, sweet sorghum transport stage, bio-ethanol production stage, and bio-ethanol transport and distribution stage, accordingly; and FE_by_ is the energy saved using by-products.

Formulas of BE, FE_i_ (*i* = 1,2,3,4) and FE_by_ are as follows [30, 31]:

$$ {\text{BE = HCV}}_{{{\text{ethanol}}}} , $$

where HCV_ethanol_ is the high calorific value of bio-ethanol, which is 29.66 MJ/kg:

$$ {\text{FE}}_{1} = \frac{{\sum\nolimits_{i} {\left( {{\text{XEI}}_{i} \times {\text{X}}_{i} } \right)} }}{{{\text{Y}}_{{{\text{sw}}}} \times \beta }}, $$

where XEI_i_ is the energy intensity of the input materials *i*, *X*_i_ is the input quantity of material *i*, *Y*_sw_ is the yield of sweet sorghum biomass, and *β* is the conversion ratio of sweet sorghum to ethanol:

$$ {\text{FE}}_{2} = \frac{{{\text{TE}}_{1} \times d_{1} \times H_{1} }}{{Y_{{{\text{sw}}}} \times \beta }}, $$

where TE_1_ is the quantity of fossil fuel input at sweet sorghum transport stage, *d*_1_ is the average distance of transporting sweet sorghum to factories, and *H*_1_ is the energy intensity of the fossil fuel:

$$ {\text{FE}}_{3} = \sum\nolimits_{i} {\left( {{\text{E}}_{i} \times {\text{EEI}}_{i} } \right)} , $$

where *E*_i_ is the quantity of energy input (e.g., electricity, coal, etc.) of bio-ethanol production in factories, and EEI_i_ is the corresponding energy intensity of E_i_:

$$ {\text{FE}}_{4} = {\text{TE}}_{2} \times d_{2} \times H_{2} , $$

where TE_2_ is the quantity of fossil fuel input at bio-ethanol transport and distribution stage, d_2_ is the average distance of transporting bio-ethanol to gasoline stations, and *H*_2_ is the energy intensity of the fossil fuel:

$$ {\text{FE}}_{by} = \sum\limits_{i} {\left( {{\text{EW}}_{i} \times {\text{M}}_{i} } \right)} , $$

where EW_i_ is the energy intensity of by-products and M_i_ is the yield of by-product.

### LC-GHG emission mitigation assessment

Before conducting LC-GHG emission mitigation assessment, the carbon balance analysis of sweet sorghum-based ethanol should be clearly presented, because carbon dioxide comes from the atmosphere at sweet sorghum planting stage, and goes back to the atmosphere through bio-ethanol combustion stage [31]. As shown in Fig. 8, inorganic carbon (carbon dioxide) from the atmosphere is assimilated into organic compounds during the process of sweet sorghum growth and development, and is released to the atmosphere through fermentation and residues of the ethanol production procedure and the course of ethanol combustion [31]. This kind of carbon forms a cycle, and a balance is maintained.

However, to guarantee the life cycle from sweet sorghum to ethanol combustion, the system requires extra carbon in forms of chemical fertilizers, fossil fuels, and so on, which are the main sources of bio-ethanol GHG emissions. The inventories of LC-GHG emission mitigation are listed in Fig. 8 (see boxes with green edges in Fig. 8). LC-GHG emission is calculated by the sum of extra carbon releases minus the avoided carbon release of by-product, and the equation is as follows:

$$ C_{{{\text{fossil}}}} = C_{1} + C_{2} + C_{3} + C_{4} - C_{{{\text{avoided}}}} , $$

where *C*_fossil_ is the GHG emission over the life cycle of sweet sorghum-based ethanol by the unit kg CO_2_ eq/t ethanol; *C*_*i*_ (*i* = 1,2,3,4) are GHG emissions of sweet sorghum at planting and harvesting stage, sweet sorghum transport stage, bio-ethanol production stage, and bio-ethanol transport and distribution stage by the unit kg CO_2_ eq/t ethanol, accordingly; *C*_avoided_ is the carbon release avoided by recycling and usage of the by-products by the unit kg CO_2_ eq/t ethanol.

The formula of LC-GHG emission mitigation is:

$$ C_{{{\text{mitigation}}}} = {\text{BE}} \times W_{{{\text{gasoline}}}} - C_{{{\text{fossil}}}} , $$

where BE is the energy output of bio-ethanol according to Sect. 6.3.1, W_gasoline_ is conversion ratio of calorific value to GHG emission of gasoline with the value of 0.0189 kg CO_2_ eq·MJ^*−*1^.

### Data preparation

#### Field experiments on sweet sorghum cultivation on saline–alkali land

Field experiments were conducted in an energy crop cultivation base in Xianhe Town, Hekou District, Dongying, in 2014. PALO ALTO Biomass Sorghum was selected for the experiments, which is a hybrid sweet sorghum variety bred by NexSteppe Company in Scottsdale, the USA. PALO ALTO Biomass Sorghum has some primary superior qualities. The growing period of this high-yielding biomass sorghum ranges from 120 to 140 days, and its water content is low. After harvest, it can be directly burned in the boiler, and its calorific value and chemical composition are consistent with the current biomass source. And the sorghum stalks provide extra straw for fertilizing the next crop. Moreover, farmers obtain excellent profitability [32].

According to the planting instructions of NexSteppe, five steps were implemented during the growth of PALO ALTO Biomass Sorghum, which included seed dressing, sowing, fertilizing, weeding, and fertilizing for a second time, as shown in Additional file 1: Appendix D. During field experiments, crop growth status was monitored and reported weekly, as shown in appendix E. The harvest date was November 4, 2014, and yield per hectare was 63,000 kg/ha.

### Data preparation for the DSSAT model

#### Geospatial dataset of saline–alkali land

Saline–alkali land is a poor-quality land type. Typically, a clay soil with pH > 8.5 or soil with high salt content is considered saline–alkali soil. In this study, four steps were used to extract the spatial distribution of saline–alkali land.

- Step 1: First, saline–alkali soil types were ascertained through relevant literature, because saline–alkali soil is not a basic classification unit in the soil classification system.
- Step 2: Second, spatial distributions of the soil types confirmed in the first step were extracted from the China Soil Database (http://www.soil.csdb.cn/map/).
- Step 3: Third, soil properties of eight different soil layers were extracted from a published soil data set GSDE (a Global Soil Data Set for Earth System Modeling), which was harmonized and processed based on the Soil Map of the World and various regional and national soil databases following an improved protocol of the Harmonized World Soil Database (HWSD) [33, 34]. GSDE provides 11 types of soil general information for soil profiles and 34 soil properties (see Additional file 1: Appendix F), and each soil property is captured in eight layers to the depth of 2.3 m.
- Step 4: Finally, dataset of saline–alkali land was carefully organized and imported to the DSSAT model.

#### Daily weather data

DSSAT model requires daily weather data available for the duration of the growing season, from the day of planting to the day of crop maturity. Ideally, to help the simulation and provide an estimation of soil conditions at planting time, beginning before planting day, and continuing to the end after crop maturity are preferred for the duration. Nine general variables and eight daily variables of weather conditions were examined, as shown in Additional file 1: Appendix G. Grid-by-grid daily solar radiation (SRAD), maximum air temperature (TMAX), minimum air temperature (TMIN), and precipitation (RAIN) were simulated by ANUSPLIN Version 4.3, which provides a facility for transparent analysis and interpolation of noisy multivariate data using thin-plate smoothing splines, through comprehensive statistical analyses, data diagnostics, and spatially distributed standard errors [35–37]. The original weather data were station monitoring data downloaded from the CMA website (http://data.cma.cn/).

#### Crop cultivar coefficients

The cultivar coefficients were localized based on the field experiment data using the GLUE (Generalized Likelihood Uncertainty Estimation) method, which is a Bayesian estimation method that uses Monte Carlo sampling from prior distributions of the coefficients and a Gaussian likelihood function to determine the best coefficients based on experimental data [38, 39]. The results of sweet sorghum cultivar coefficients are shown in Table 3.

**Table 3:** Sweet sorghum cultivar coefficients

Coefficient	Definitions^a^	Cultivar
P1	Thermal time from seedling emergence to the end of the juvenile phase (expressed in degree days above TBASE during which the plant is not responsive to changes in photoperiod)	403.5
P2O	Critical photoperiod or the longest day length (in hours) at which development occurs at a maximum rate. At values higher than P2O, the rate of development is reduced	102.0
P2R	Extent to which phasic development leading to panicle initiation (expressed in degree days) is delayed for each hour increase in photoperiod above P2O	12.92
P5	Thermal time from beginning of grain filling to physiological maturity (degree days above TBASE)	191.3
G1	Scaler for relative leaf size	617.5
G2	Scaler for partitioning of assimilates to the panicle (head)	436.6
PHINT	Phylochron interval; the interval in thermal time between successive leaf tip appearances (degree days)	228.8
P3	Thermal time from the end of flag leaf expansion to anthesis (degree days above TBASE)	553.4
P4	Thermal time from anthesis to beginning of grain filling (degree days above TBASE)	49.00
P2	Thermal time from the end of the juvenile stage to tassel initiation under short days (degree days above TBASE)	8.864
PANTH	Thermal time from the end of tassel initiation to anthesis (degree days above TBASE)	6.051

^a^Definitions are the solidified document in GLUE model

### Inventory datasets for life cycle assessment

#### Inventory analysis of LC-NEG assessment

Inventories of LC-NEG assessment are sorted out from literatures and field experimental data [30]. At sweet sorghum planting stage, the input quantities of fertilizers, herbicide, insecticide, and diesel oil were converted to energy input; see Table 4.

**Table 4:** Energy input, including the fertilizer and energy, at sweet sorghum planting stage

Item	Unit	Input (unit/ha)	Energy intensity (MJ/unit) [30]	Energy input (MJ/ha)
Nitrogen fertilizer	Kg	54	46.50	2511.00
Phosphate fertilizer	Kg	81	7.03	269.43
Potassium fertilizer	Kg	67.5	6.85	462.38
Herbicide	Kg	3.56	266.56	948.95
Insecticide	Kg	0.75	284.82	213.62
Diesel oil	L	67	44.13	2956.71
Summary				7662.08

At the stages of transport of sweet sorghum and bio-ethanol, the energy inputs of road transport and railway transport were calculated, see Table 5.

**Table 5:** Energy input at the stages of transporting sweet sorghum and bio-ethanol

Items		Sweet sorghum transport stage^a^	Bio-ethanol transport stage^b^	
Transport method		Road	Road	Railway
Distance (km)		88	100	500
Energy intensity (MJ/t km) [30]		2.21	2.21	0.077
Energy input	(MJ/t sweet sorghum)	194.48	221	38.5
	(MJ/t ethanol)	311.68	3376	616

^a^The main mode of transportation in sweet sorghum transport stage is road

^b^The main mode of transportation in bio-ethanol transport stage is combination of both road and railway

At the stage of bio-ethanol production, the energy input of electricity, steam, coal, and so on were calculated; see Table 6.

**Table 6:** Energy input at the stage of bio-ethanol production stage

Procedure or Item	Energy input				Energy avoided
	Electricity (kWh/t ethanol) [30, 40–42]	Coal (kg/t ethanol) [30, 40–42]	Steam (t/t ethanol) [30]	Other input^a^ (t/t ethanol) [30]	By-product^b^ (t/t ethanol) [30]
Pretreatment	95	–	–	–	–
Fermentation	50	–	0.2	–	–
Rectification	25	–	2.4	–	–
Dehydration	40	–	1.9	–	–
Residue handling	143	–	–	49.88	–
Accessory equipment	20	611.2	–	–	–
By-product production	106	–	–	–	–1.18
Denaturing	7.416	–	–	–	–
Quantity sum	486.42	611.2	4.5	49.88	–1.18
Energy intensity (MJ/t)	3.6	29.27	2637.61	98.70	14,670.00
Energy sum (MJ/t ethanol)	1751.10	17,889.82	11,869.25	4923.58	17,310.60
Summary	19,123.15				

^a^Other energy input mainly includes hot air

^b^By-product mainly refers to the solid granular fuel produced in the process of bio-ethanol production

### Carbon emission factors

Fossil fuel-based substances were converted to GHG emissions using conversion coefficients and carbon emission conversion coefficients from the literature [31, 40–42]. Conversion coefficients used in this article are shown in Table 7.

**Table 7:** Conversion coefficients of substances and carbon

Substance	Carbon emission factor [31, 40–42]	Unit
Nitrogen fertilizer	0.858	kg CO_2_ eq /kg
Phosphate fertilizer	0.17	kg CO_2_ eq /kg
Potassium fertilizer	0.12	kg CO_2_ eq /kg
Herbicide	4.70	kg CO_2_ eq /kg
Insecticide	4.93	kg CO_2_ eq /kg
Diesel oil	0.85	kg CO_2_ eq /L
Electricity	0.36	kg CO_2_ eq /kWh
Coal	0.52	kg CO_2_ eq /kg

### Inventory analysis of LC-GHG emission mitigation assessment

According to details of the field experiment in the section “Field experiments on sweet sorghum cultivation on saline–alkali land”, sweet sorghum planting procedures that correlated with carbon included the input of nitrogen fertilizer, phosphate fertilizer, potassium fertilizer, herbicide, insecticide, and diesel oil. To convert to carbon emissions, these input materials were multiplied by the carbon emission factor in Table 8.

**Table 8:** GHG emissions at sweet sorghum planting stage

Item	Input [40–42]	Carbon emission factor [31, 40–42]	GHG emissions (kg CO_2_ eq/ha)^a^
Nitrogen fertilizer	54 kg/ha	0.858 kg CO_2_ eq /kg	46.33
Phosphate fertilizer	81 kg/ha	0.17 kg CO_2_ eq /kg	13.77
Potassium fertilizer	67.5 kg/ha	0.12 kg CO_2_ eq /kg	8.1
Herbicide	3.56 kg/ha	4.7 kg CO_2_ eq /kg	16.73
Insecticide	0.75 kg/ha	4.93 kg CO_2_ eq /kg	3.70
Diesel oil	67 L/ha	0.85 kg CO_2_ eq /L	56.95
Summary			145.58

^a^GHG emissions are the inputs times carbon emission factors

Two stages of transport occur: one stage is sweet sorghum transported to factories, and the other is ethanol transported to stations. Two types of transport were road and railway transport, and in this study, sweet sorghum was transported by road for 88 km, whereas ethanol was transported by the combination of road transport for 100 km and railway transport for 500 km. In China, there are 38% trains with an internal-combustion engine and 62% trains with electricity. The carbon emission analysis is shown in Tables 9 and 10.

**Table 9:** GHG emission analysis of road transport

Stage	Distance (km)	Fuel intensity (L·t^−1^·km^−1^) [40–42]	Carbon emission factor [31, 40–42]	Conversion coefficient to ethanol	GHG emission (kg CO_2_ eq/t ethanol)^a^
Sweet sorghum transport	88	0.05	0.85 kg CO_2_ eq /L	16	59.84
Ethanol transport	100	0.05	0.85 kg CO_2_ eq /L	1	4.25
Summary	188	–	–	–	64.09

^a^ GHG emissions are the energy inputs of transportation times carbon emission factors and conversion coefficient

**Table 10:** GHG emission analysis of railway transport

Transport method	Distance (km)	Fuel intensity [40–42]	Carbon emission factor [31, 40–42]	Percentage	GHG emission (kg CO_2_ eq /t ethanol)^a^
Railway transport with diesel	500	3.27 L/(thousand t·km)	0.85 kg CO_2_ eq/L	38%	1.39
Railway transport with electricity	500	11.2 kWh/(thousand t·km)	0.26 kg CO_2_ eq/kWh	62%	1.456
Summary	–	–	–	100%	1.43

^a^GHG emissions are the energy inputs of transportation times carbon emission factors and the proportion of transportation mode

At the ethanol production stage, the consumption of electricity and coal was the basis for analysis of GHG emissions. The GHG emission analysis of ethanol production is shown in Table 11.

**Table 11:** GHG emission analysis of ethanol production

Procedure or Item	Energy type	
	Electricity [30, 40–42]	Coal [30, 40–42]
Pretreatment	95 kWh/t ethanol	–
Fermentation	50 kWh/t ethanol	–
Rectification	25 kWh/t ethanol	–
Dehydration	40 kWh/t ethanol	–
Residue handling	143 kWh/t ethanol	–
Accessory equipment	20 kWh/t ethanol	611.2 kg/t ethanol
By-product production	106 kWh/t ethanol	–
Denaturing	7.416 kWh/t ethanol	–
Avoided by by-products	− 106 kWh/t ethanol	–
Energy consumption	380.416 kWh/t ethanol	611.2 kg/t ethanol
Carbon coefficients	0.26 kg CO_2_ eq /kWh	0.52 kg CO_2_ eq /kg coal
Carbon emission	98.91 kg CO_2_ eq/t ethanol	317.824 kg CO_2_ eq/t ethanol

After ethanol being transported to stations, electricity is the primary power source, and in this study, the energy consumption was 0.0007 kWh/L, equivalent to 0.32 kg CO_2_ eq/t ethanol.

## Supplementary Information

**Additional file 1: Appendix A.** Appendices Study area. **Appendix B.** Data for the histogram in Fig. 3. **Appendix C.** Data for the histogram in Fig. 5. **Appendix D.** Crop management of field experiments on PALO ALTO Biomass Sorghum. **Appendix E.** Status of crop growth during field experiment. **Appendix F.** Spatial soil profile properties prepared for DSSAT model. **Appendix G.** Variables of daily weather data.

## Data Availability

Availability of data and materials have been stated in the section “Data preparation”. Please refer to the specific part for the hyperlinks of data sources.
