# Supplementary material for: Assessing the sweet sorghum-based ethanol potential on saline–alkali land with DSSAT model and LCA approach
Source: Biotechnol Biofuels. 2021 Feb 16;14:44. doi: 10.1186/s13068-021-01896-z (PMC7885580; doi:10.1186/s13068-021-01896-z)
Supplement: Supplementary file 1 — Additional file 1: Appendix A. Appendices Study area. Appendix B. Data for the histogram in Fig. 3. Appendix C. Data for the histogram in Fig. 5. Appendix D. Crop management of field experiments on PALO ALTO Biomass Sorghum. Appendix E. Status of crop growth during field experiment. Appendix F. Spatial soil profile properties prepared for DSSAT model. Appendix G. Variables of daily weather data. [file 13068_2021_1896_MOESM1_ESM.doc]

1. Appendices Study area


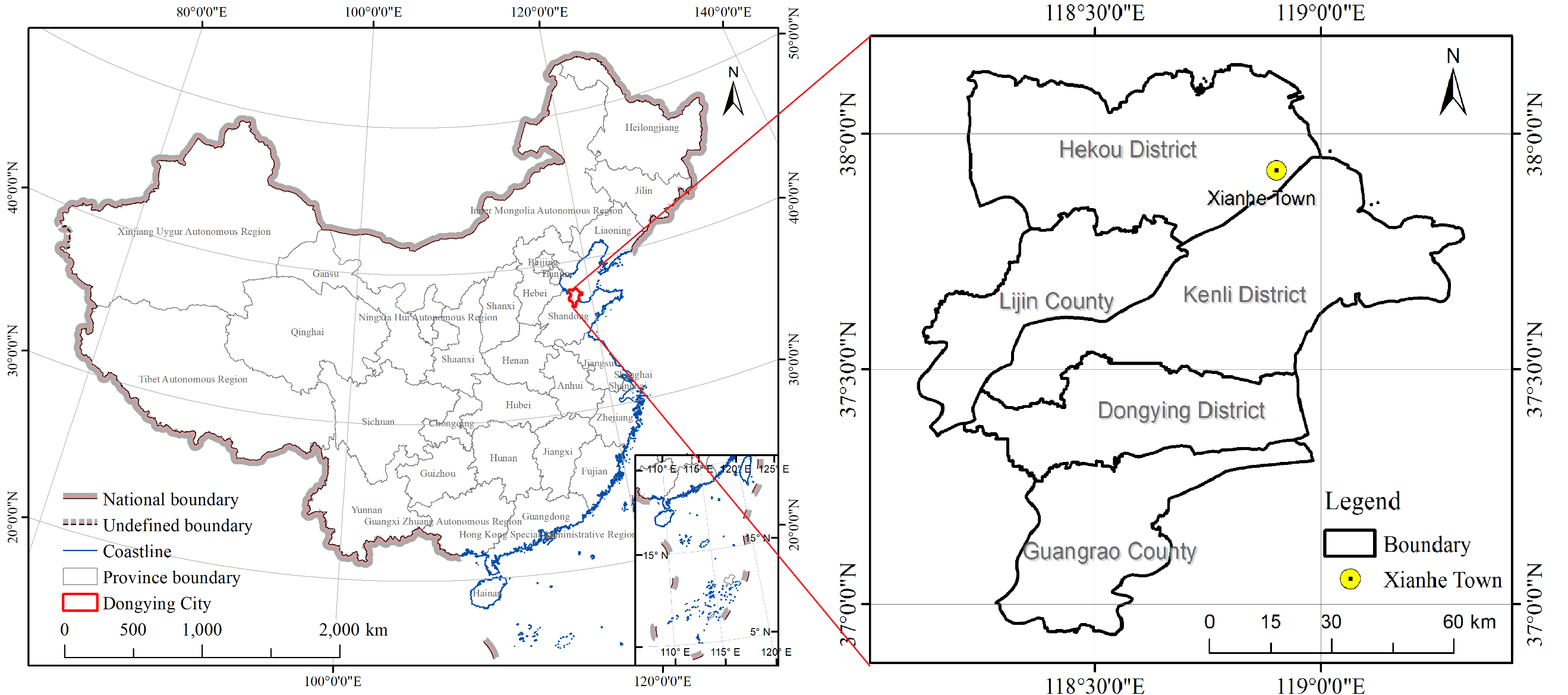


1. Data for the histogram in Fig.3

| GHG emission mitigation range  (t CO_2_ eq/km^2^) | Area (km^2^) | Cumulative Sum (km^2^) | Cumulative Percent (%) |
| --- | --- | --- | --- |
| 8 to 8.49 | 1 | 1 | 0.02 |
| 8.5 to 8.99 | 2 | 3 | 0.07 |
| 9 to 9.49 | 5 | 8 | 0.19 |
| 9.5 to 9.99 | 12 | 20 | 0.47 |
| 10 to 10.49 | 8 | 28 | 0.66 |
| 10.5 to 10.99 | 37 | 65 | 1.53 |
| 11 to 11.49 | 44 | 109 | 2.56 |
| 11.5 to 11.99 | 75 | 184 | 4.32 |
| 12 to 12.49 | 140 | 324 | 7.60 |
| 12.5 to 12.99 | 170 | 494 | 11.59 |
| 13 to 13.49 | 281 | 775 | 18.19 |
| 13.5 to 13.99 | 228 | 1003 | 23.54 |
| 14 to 14.49 | 329 | 1332 | 31.26 |
| 14.5 to 14.99 | 511 | 1843 | 43.25 |
| 15 to 15.49 | 877 | 2720 | 63.83 |
| 15.5 to 15.99 | 620 | 3340 | 78.39 |
| 16 to 16.49 | 308 | 3648 | 85.61 |
| 16.5 to 16.99 | 91 | 3739 | 87.75 |
| 17 to 17.49 | 207 | 3946 | 92.61 |
| 17.5 to 17.99 | 315 | 4261 | 100.00 |
| 18 to 18.49 | 0 | 4261 | 100.00 |

1. Data for the histogram in Fig.5

| NEG range  (million MJ/km2) | Area  (km2) | Cumulative Sum  (km2) | Cumulative Percent  (%) |
| --- | --- | --- | --- |
| 0.55 to 0.599 | 2 | 2 | 0.05 |
| 0.6 to 0.649 | 4 | 6 | 0.14 |
| 0.65 to 0.699 | 7 | 13 | 0.31 |
| 0.7 to 0.749 | 13 | 26 | 0.61 |
| 0.75 to 0.799 | 10 | 36 | 0.84 |
| 0.8 to 0.849 | 63 | 99 | 2.32 |
| 0.85 to 0.899 | 68 | 167 | 3.92 |
| 0.9 to 0.949 | 145 | 312 | 7.32 |
| 0.95 to 0.999 | 182 | 494 | 11.59 |
| 1 to 1.049 | 300 | 794 | 18.63 |
| 1.05 to 1.099 | 250 | 1044 | 24.50 |
| 1.1 to 1.149 | 368 | 1412 | 33.14 |
| 1.15 to 1.199 | 666 | 2078 | 48.77 |
| 1.2 to 1.249 | 987 | 3065 | 71.93 |
| 1.25 to 1.299 | 442 | 3507 | 82.30 |
| 1.3 to 1.349 | 206 | 3713 | 87.14 |
| 1.35 to 1.399 | 134 | 3847 | 90.28 |
| 1.4 to 1.449 | 397 | 4244 | 99.60 |
| 1.45 to 1.499 | 17 | 4261 | 100.00 |
| 1.5 to 1.549 | 0 | 4261 | 100.00 |

1. Crop management of field experiments on PALO ALTO Biomass Sorghum

| Operation | Operation time | Details |
| --- | --- | --- |
| Seed dressing | May 28, 2014 | Non-systemic fungicide Fludioxoni 0.1 ml/kg;  Systemic fungicide Metalaxyl 0.265 ml/kg;  Systemic insecticide Thiamethoxam 4 ml/kg. |
| Sowing | May 29, 2014 | Row spacing: 50 cm;  Spacing in the rows: 10 cm;  Ditch depth: 10 cm;  Soil thickness: 2-3 cm;  Seed number: 16,000. |
| Fertilizing | May 29, 2014 | Compound fertilizer 450 kg/ha; content of N-P-K 12-18-15%. |
| Weeding | June 1, 2014 | Spray Atrazine 3 L/ha. |
| Topdressing | June 25, 2014 | Compound fertilizer 450 kg/ha; content of N-P-K 12-18-15%. |

1. Status of crop growth during field experiment

| Week | Period | Total number planted | Total number emerged | Emergence rate | Height of strong plantlets (cm) | Height of common plantlets (cm) |
| --- | --- | --- | --- | --- | --- | --- |
| 1 | 6.2-6.8 | 16000 | 11200 | 0.7 |  |  |
| 2 | 6.9-6.15 | 16000 | 13600 | 0.85 | 13 |  |
| 3 | 6.16-6.22 | 16000 | 14400 | 0.9 | 25 |  |
| 4 | 6.23-6.29 | 16000 | 15200 | 0.95 | 60 | 20 |
| 5 | 6.30-7.6 | 16000 | 15200 | 0.95 | 110 | 27 |
| 6 | 7.7-7.13 | 16000 | 15200 | 0.95 | 140 | 68 |
| 7 | 7.14-7.20 | 16000 | 15200 | 0.95 | 173 | 125 |
| 8 | 7.21-7.27 | 16000 | 15200 | 0.95 | 180 | 125 |
| 9 | 7.28-8.3 | 16000 | 15200 | 0.95 | 205 | 165 |
| 10 | 8.4-8.10 | 16000 | 15200 | 0.95 | 215 | 175 |
| 11-12 | 8.11-8.24 | 16000 | 15200 | 0.95 | 240 | 220 |
| 13-14 | 8.25-9.7 | 16000 | 15200 | 0.95 | - | - |
| 15-16 | 9.8-9.21 | 16000 | 15200 | 0.95 | - | - |
| 17-19 | 9.22-10.12 | 16000 | 15200 | 0.95 | - | - |
| 20-21 | 10.13-10.26 | 16000 | 15200 | 0.95 | 450 | 350 |

1. Spatial soil profile properties prepared for DSSAT model

| Attribute | Default value | Unit | Scale factor |
| --- | --- | --- | --- |
| Total carbon | 0 | % of weight | 0.01 |
| Organic carbon | 0 | of weight | 0.01 |
| Total N | 0 | % of weight | 0.01 |
| Total S | 0 | % of weight | 0.01 |
| CaCO3 | 0 | % of weight | 0.01 |
| Gypsum | 0 | % of weight | 0.01 |
| pH (H2O) | 70 |  | 0.1 |
| pH (KCl) | 70 |  | 0.1 |
| pH (CaCl2) | 70 |  | 0.1 |
| Electrical conductivity | 600 | ds/m | 0.01 |
| Exchangeable calcium | 0 | cmol/kg | 0.01 |
| Exchangeable magnesium | 0 | cmol/kg | 0.01 |
| Exchangeable sodium | 0 | cmol/kg | 0.01 |
| Exchangeable potassium | 0 | cmol/kg | 0.01 |
| Exchangeable aluminum | 0 | cmol/kg | 0.01 |
| Exchangeable acidity | 0 | cmol/kg | 0.01 |
| Cation exchange capacity | 0 | cmol/kg | 0.01 |
| Base saturation | 0 | % |  |
| Sand contentb | 50 | % of weight |  |
| Silt content | 30 | % of weight |  |
| Clay content | 20 | % of weight |  |
| Gravel content | 0 | % of volume |  |
| Bulk density | 120 | g/cm3 | 0.01 |
| Volumetric water content at -10 kPa | 35 | % of volume |  |
| Volumetric water content at -33 kPa | 30 | % of volume |  |
| Volumetric water content at -1500 kPa | 10 | % of volume |  |
| The amount of phosphorous using the Bray1 method | 0 | ppm of weight | 0.01 |
| The amount of phosphorous by Olsen method | 0 | ppm of weight | 0.01 |
| Phosphorous retention by New Zealand method | 0 | % of weight | 0.01 |
| The amount of water soluble phosphorous | 0 | ppm of weight | 0.0001 |
| The amount of phosphorous by Mehlich method | 0 | ppm of weight | 0.01 |
| Exchangeable sodium percentage | 0 | % of weight | 0.01 |
| Total phosphorus | 0 | % of weight | 0.0001 |
| Total potassium | 0 | % of weight | 0.01 |

1. Variables of daily weather data

| No. | Variable name | Variable | Unit | Note |
| --- | --- | --- | --- | --- |
| 1 | INSTE | Institute code |  |  |
| 2 | SITEE | Site code |  |  |
| 3 | XLAT | Latitude | Degrees (decimals) |  |
| 4 | XLONG | Longitude | Degrees (decimals) |  |
| 5 | ELEV | Elevation | m |  |
| 6 | TAV | Air temperature average | Degree Celsius |  |
| 7 | TAMP | Air temperature amplitude, monthly averages | Degree Celsius |  |
| 8 | REFHT | Height of temperature measurements | M |  |
| 9 | WNDHT | Height of wind measurements | M |  |
| 10 | YRDOYW | Year+days from Jan. 1 |  |  |
| 11 | SRAD | Solar radiation | MJ·m^-2^·day^-1^ |  |
| 12 | TMAX | Maximum air temperature | Degree Celsius |  |
| 13 | TMIN | Minimum air temperature | Degree Celsius |  |
| 14 | RAIN | Precipitation | Mm |  |
| 15 | TDEW | Dew point temperature | Degree Celsius | Optional |
| 16 | WINDSP | Wind run | km·day^-1^ | Optional |
| 17 | PAR | Photosynthetic active radiation | moles·m^-2^·day^-1^ | Optional |
